# Correlation between disease severity indices and quality of life measurement tools in atopic dermatitis patients

**DOI:** 10.7705/biomedica.6998

**Published:** 2024-08-29

**Authors:** Gloria Sanclemente, Natalia Hernández, Liliana Tamayo, Daniela Chaparro, Ángela López

**Affiliations:** 1 Grupo de Investigación Dermatológica, Universidad de Antioquia, Medellín, Colombia y Grupo de Innovación y Desarrollo en Investigación - CIC, Medellín, Colombia Universidad de Antioquia Grupo de Investigación Dermatológica Universidad de Antioquia Colombia y Grupo de Innovación y Desarrollo en Investigación - CIC Medellín Colombia; 2 Dermatologia, Hospital San Vicente Fundación-IPS Universitaria, Universidad de Antioquia, Medellín, Colombia Universidad de Antioquia Dermatologia Hospital San Vicente Fundación-IPS Universitaria Universidad de Antioquia Medellín Colombia; 3 Dermatosoluciones SAS, Bogotá, D.C., Colombia Dermatosoluciones SAS Dermatosoluciones SAS Bogotá, D.C. Colombia; 4 Seccion de Dermatología, Universidad Pontificia Bolivariana, Medellín, Colombia Universidad Pontificia Bolivariana Seccion de Dermatología Universidad Pontificia Bolivariana Medellín Colombia; 5 Seccion de Dermatología, Pontificia Universidad Javeriana, Bogotá, D.C., Colombia Pontificia Universidad Javeriana Seccion de Dermatología Pontificia Universidad Javeriana Bogotá, D.C. Colombia; 6 IPS Fototerapia Bojanini y López SAS. Bogotá, D.C., Colombia IPS Fototerapia Bojanini y López SAS IPS Fototerapia Bojanini y López SAS Bogotá, D.C. Colombia

**Keywords:** Dermatitis, atopic, correlation of data, quality of life, patient acuity, dermatitis atópica, correlación de datos, calidad de vida, gravedad del paciente

## Abstract

**Introduction.:**

Reports regarding the correlation and effect size of change of the full spectrum of quality of life and disease severity measures applied in-person to patients with atopic dermatitis are scarce.

**Objectives.:**

To assess quality-of-life with 3 different instruments and to evaluate disease severity indices and to determine their correlation and effect size of change between two measurements.

**Materials and methods.:**

Patient-level data were obtained through two in-person visits. Sociodemographic information and data related to disease distribution, severity (through the BSA, EASI, SCORAD, POEM, and itching scales), and the impact of atopic dermatitis on quality of life using the DLQI and Skindex-29, and EQ-5D, were assessed. The correlation between change in quality-of-life scores and disease severity scores in addition to the standardized effect size were also evaluated.

**Results.:**

Only 139 out of 212 patients completed the follow-up visit. BSA highly correlated with SCORAD and EASI, and the lowest correlation was found with POEM. The best correlation of pruritus VAS was found with sleep disturbance. The SCORAD score highly correlated with EASI, and the lowest correlation was found with POEM. The magnitude of the effect at initiation of the study vs follow-up was in average moderate to important.

**Conclusions.:**

Patients with atopic dermatitis experience a substantial burden on quality of life. Disease activity correlates better with quality-of-life measurements when the disease is less severe after starting therapy. POEM and Skindex-29 seem to be optimal to determine disease severity and quality of life in adults with atopic dermatitis.

## Resumen

**Introducción.:**

La información publicada sobre la correlación entre la magnitud del efecto de todo el espectro de la calidad de vida y la gravedad de la enfermedad en pacientes con dermatitis atópica es escasa.

**Objetivos.:**

Evaluar la calidad de vida con tres instrumentos diferentes y los índices de gravedad de la enfermedad en pacientes con dermatitis atópica para determinar su correlación y el tamaño del efecto del cambio.

**Materiales y métodos.:**

Los datos de los pacientes se obtuvieron a partir de dos visitas. Se evaluó la información sociodemográfica y los datos relacionados con la distribución y la gravedad de la enfermedad (mediante de las escalas BSA, EASI, SCORAD, POEM, prurito) y el impacto de la dermatitis atópica en la calidad de vida utilizando el *Dermatology Life Quality Index*, Skindex-29 y EQ-5D. También se evaluó la correlación entre el cambio en las puntuaciones de calidad de vida y las de gravedad de la enfermedad, además del tamaño del efecto estandarizado.

**Resultados.:**

Solo 139 de los 212 pacientes completaron la visita de seguimiento. El área de superficie corporal se correlacionó fuertemente con el SCORAD y el EASI, y la correlación más débil fue con el POEM. La mejor correlación del prurito medido con la escala visual análoga se halló con la alteración del sueño. El puntaje SCORAD se correlacionó altamente con el EASI mientras que la correlación más baja se encontró con el POEM. La magnitud del efecto al inicio del estudio respecto al seguimiento fue en promedio de moderada a importante.

**Conclusiones.:**

Los pacientes con dermatitis atópica experimentan una carga sustancial en la calidad de vida. La actividad de la enfermedad se correlaciona mejor con las mediciones de calidad de vida cuando esta es menos grave, después de comenzar la terapia. Los índices POEM y Skindex-29 parecen ser óptimos para determinar la gravedad de la enfermedad y la calidad de vida en adultos con dermatitis atópica.

Atopic dermatitis is a chronic distressing inflammatory skin disease characterized by intense pruritus, a waxing and waning course, and multiple comorbidities such as asthma and allergic rhinitis, all of which can lead to significant morbidity ^1^^-^^3^. It most often presents in early childhood and can persist throughout adulthood. It can appear at any age and compromise any gender and ethnicity ^4^^,^^5^. The prevalence of atopic dermatitis has increased markedly in the United States over the past five decades, with current reports ranging from 10 to 20% in American children. It has quickly become a public health problem due to its high prevalence, especially in industrialized countries, and due to the high cost and economic burden that it implies ^4^^,^^6^^,^^7^.

The chronic and relapsing nature of the disease greatly affects the wellbeing, academic and occupational performance, and quality of life of patients and their families, especially in those with moderate or severe forms ^8^. Instruments used in clinical trials to measure the quality of life and disease severity in atopic dermatitis include the Dermatology Life Quality Index (DLQI), and the Body surface area (BSA), Eczema Area and Severity Index (EASI), Scoring AD (SCORAD) and Patient Oriented Eczema Measure (POEM), respectively. Such tools validated and standardized by the Harmonizing Outcome Measures for Eczema (HOME) ^9^, in addition to the Skindex-29, have been reported to be useful for physicians and health insurers for shared decision-making and deciding among several therapeutic options ^10^^,^^11^.

Previous reports do not cover the full spectrum of quality of life and disease severity measures applied at the same visit to all patients. Data regarding the correlation between disease severity and quality of life scales is intriguing and scarce. Therefore, the aim of this study was to assess the quality of life in atopic dermatitis Colombian patients with three different instruments (DLQI, Skindex-29, and EQ-5D) and disease severity indices such as the pruritus visual analogue scale (VAS), body surface area (BSA), eczema area and severity Index (EASI), scoring atopic dermatitis (SCORAD), and patient oriented eczema measure (POEM) to determine their probable correlation.

## Materials and methods

Patient-level data were obtained within the framework of a multicenter nationwide cross-sectional study of atopic dermatitis. Colombian patients older than 12 years or foreigners residing for more than 15 years in the country with a clinical diagnosis of moderate to severe atopic dermatitis were included. Patients with any mental disability were excluded.

Patients attended different health provider institutions, hospitals, and dermatology private practice offices in six different Colombian regions: northwest (main city: Barranquilla), northeast (main city: Bucaramanga), central area (main cities: Bogotá, Medellín, Armenia); southwest (main city: Cali). Informed consent was obtained from all adult patients or from all children and their parents or guardians.

The data presented in this study included two visits: One initial visit (initial) performed before the COVID-19 pandemic (until February 2020), for which epidemiologic features have already been published ^12^, and a second visit, performed at the end of 2020 until the first semester of 2021. At both inperson visits, all trained investigators assessed sociodemographic information and data related to disease distribution, severity (applying BSA, EASI, SCORAD, and POEM scales), and the impact of atopic dermatitis on quality of life using the DLQI, EQ-5D and Skindex-29.

Itching intensity was measured with the visual analog scale (VAS) categorized according to Reich *et al.*^13^, as follows: 0 = no pruritus, > 0 to < 4 points = mild pruritus, ≥ 4 to < 7 points = moderate pruritus, ≥ 7 to < 9 points = severe pruritus, and ≥ 9 points = very severe pruritus. Sleep disturbance was also evaluated with the visual analog scale (VAS). The percentage of BSA involved was classified as clear (0%), mild (> 0.1 to < 16%), moderate (16 to < 40%), and severe (40 to 100%), as previously described ^14^. The EASI strata were categorized as follows: 0 = clear; 0.1 to 1 = almost clear; 1.1 to 7 = mild; 7.1 to 21 = moderate; 21.1 to 50 = severe; 50.1 to 72 = very severe as previously described ^15^. The SCORAD strata were classified as clear-mild = 0 to 28.9, moderate = 29 to 48.9, and severe = 49 to 103, according to Chopra *et al.*^16^. POEM is a seven-item questionnaire that measures the frequency of child symptoms, including itch, bleeding, sleep disturbance, weeping or oozing, and flaking, cracked, and dry skin. Each question was scored from 0 = ‘No days’ to 4 = ‘Every day’ for a maximum score of 28. The severity banding for the POEM score was classified as previously described by Charman *et al.*^17^: 0 - 2 = clear/almost clear, 3 - 7 = mild, 8 -16 = moderate, 17 - 24 = severe, and 25 - 28 = very severe atopic eczema.

For measuring the quality of life, we used the EQ-5D, a standardized generic instrument consisting of two parts: EQ-VAS records the respondents’ self-rated health on a visual analogue scale, from 0 to 100 (worst to best imaginable health state). Health status was measured considering the following dimensions: Mobility, self-care, usual activities, pain/discomfort, and anxiety/depression. Each health dimension has three response levels: ‘no problems’, ‘some problems’, and ‘extreme problems’. EQ-5D reflects the current general health status. Other quality-of-life instruments used were the Colombian-Spanish dermatology life quality index (DLQI) and the Colombian- validated version of Skindex-29. The DLQI is a 10-question survey: scores range from 0 to 30, with higher scores indicating worse quality of life. DLQI scoring was classified according to Hongbo *et al.*^18^ as follows: 0 - 1 = ‘no effect at all on patient’s life’, 2 - 5 = ‘small effect on patient’s life’, 6 - 10 = ‘moderate effect on patient’s life’, 11 - 20 = ‘very large effect on patient’s life’, 21 - 30 = ‘extremely large effect on patient’s life’. The Colombian version of Skindex-29 has 29 questions split into three domains: Symptoms, emotions, and functioning, with a higher score also indicating a worse quality of life. Skindex-29 cutoffs for mild (≥ 20), moderate (≥ 30), and severe (≥ 40) impairment were those suggested by Prinsen *et al*^19^.

The assessment of the quality of life and the disease severity correlation was an *a priori* secondary objective of the study in patients who fulfilled the two in-person visits.

### Statistical analysis

The frequency of the variables was described by performing a univariate analysis. The mean and standard deviation were used for continuous quantitative variables fulfilling the assumption of normality by the Kolmogorov- Smirnov test. The median, the interquartile range, and minimum and maximum values were used for those variables not fulfilling the normal distribution. For qualitative variables, absolute and relative frequencies and percentages were presented.

The correlation between the change in the quality-of-life scores and the change in the disease severity scores was analyzed using Spearman (r) or Pearson coefficient with their 95% confidence interval (CI), as required, for all combinations of the quality-of-life measurement tools and the disease severity indices (pruritus VAS, BSA, EASI, SCORAD, and POEM). Correlation coefficient cutoffs were the absolute value of 0.0 - 0.19 for ‘very weak’, 0.20 - 0.39 for ‘weak’, 0.40 - 0.59 for ‘moderate’, 0.60 - 0.79 for ‘strong’, and 0.80 - 1.0 for ‘very strong’, as described by Evans *et al.*^20^.

To test the significance of the differences between initial measurements versus the follow-up visit, we used the Student t test for dependent variables. To assess the relevance of changes between one measurement (initial) and the other (follow-up) in all comparisons, we calculated the standardized effect size using Cohen’s D with a 95% CI. Values < 0.20 reflected ‘no change’; 0.20 to 0.50, ‘slight change’; 0.50 to 0.80, ‘moderate change’, and > 0.80, ‘important or large change’. The SPSS (Statistical Package for Social Sciences), version 28, was used for data processing. The significance level was set at 0.05 for all tests.

This study was conducted in accordance with the declaration of Helsinki and approved by the institutional review board of the *Hospital San Vicente Fundaci*ó*n* and of the *IPS Universitaria, Universidad de Antioquia*, at Medellin (Colombia).

## Results

From 212 patients enrolled, only 139 patients (65.5%); 57 (41%) men and 82 (59%) women completed the follow-up visit. Non-responders had similar sociodemographic features to responders (data not shown). Patients' characteristics are summarized in table 1.

**Table 1:** Patient characteristics (N = 139)

Variable		
Sex (female) [n (%)]		82 (59)
Age (years) [mean ± SD)]		29.1 ± 13.6
Marital status [n (%)]		
	Single	100 (71.9)
	Married	31 (22.3)
	Living common-law	4 (2.9)
	Divorced	4 (2.9)
Social security classification [n (%)]		
	Contributory	103 (74.1)
	Subsidized	15 (10.8)
	Special regime	8 (5.8)
	Private	13 (9.4)

The sociodemographic characteristics of the included patients at baseline had previously been published ^12^. The mean study duration per patient (time between the initial and the follow-up visit) was 16 months (SD ± 3; range: 13 - 21 months). Most patients (76 %) started therapy with topical or systemic drugs after the initial visit.

Average disease severity at the initial visit was moderate, assessed by the BSA, EASI, SCORAD, and POEM scores, and it improved at the follow-up visit (table 2). All before and after comparisons of disease severity scales were statistically significant (p < 0.001; Student t test for dependent variables).

**Table 2:** Mean and median values of disease severity scores at the initial and the follow-up visit

	Pruritus visual analogue scale	Sleep disturbance visual analogue scale	BSA	EASI	SCORAD	POEM
Initial Mean ± SD	6.39 ± 2.9	5.11 ± 3.2	33.6 ± 25.6	13.01 ± 10.7	43.87 ± 19.35	8.9 ± 6.2
Median	7.00	7.00	26.2	9.65	44.35	8.00
Follow-up						
Mean ± SD	3.71 ± 2.6	2.3 ± 2.92	13.58 ± 14.8	4.32 ± 5.38	20.93 ± 15.1	3.03 ± 1.2
Median	3.00	1.00	9.00	2.0	18.4	4.00

BSA: Body surface area; EASI: Eczema Area and Severity Index; SCORAD: Scoring Atopic Dermatitis; POEM: Patient Oriented Eczema Measure.

### Correlations between atopic dermatitis severity scores: BSA, SCORAD, EASI and POEM

Analysis of the correlations showed that BSA was the only score highly correlated with the SCORAD (Pearson's correlation = 0.70; 95% CI = 0.61 - 0.78) and EASI indices (Pearson's correlation = 0.74; 95% CI = 0.66 - 0.81). The lowest correlation was found with POEM (Pearson's correlation = 0.20; 95% CI = 0.03 - 0.36). The best correlation of pruritus VAS was found with sleep disturbance VAS (Pearson's correlation = 0.69 (95% CI = 0.59 - 0.77).

The SCORAD score highly correlated with the EASI (Pearson's correlation = 0.65; 95% CI = 0.55 - 0.74). The lowest correlation was found with POEM (Pearson's correlation = 0.29; 95% CI = 0.13 - 0.44).

### Quality of life

Moderate to severe quality of life impairment was found at the initial visit, but at the follow-up visit, all assessments improved (table 3). All before and after quality-of-life scale comparisons were statistically significant (p < 0.001; Student t test for dependent variables). The magnitude of the effect at the initial visit versus the follow-up was, on average, moderate to important (table 4).

**Table 3:** Mean and median values of quality-of-life scores

	DLQI	EQ-5D index value	EuroQoL visual analogue scale (EQ-VAS)	Skindex-29 Total	Skindex-29 Symptoms	Skindex-29 Emotions	Skindex-29 Function
Initial Mean ± SD	9.14 ± 8.1	0.86 ± 0.11	64 ± 22.5	50.63 ± 21.57	61.81 ± 21.37	54.42 ± 24.87	40.95 ± 24.88
Median	7.00	0.88	70	50.00	60.71	55	39.58
Follow-up Mean ± SD	5.09 ± 4.8	0.94 ± 0.08	81 ± 16.9	31.65 ± 18.16	40.50 ± 20.40	35.02 ± 21.05	23.69 ± 20.15
Median	4.0	1.0	90	31.89	35.71	35.00	18.75

DLQI: Dermatology Life Quality Index

**Table 4:** Score differences of quality-of-life scores and disease severity scales between the initial and the follow-up visit

Comparisons	Cohen´s Deffect size	95%	confidence interval
EASI^1^ - EASI^2^	0.74	0.52	0.96
SCORAD^1^ - SCORAD^2^	1.09	0.84	1.33
EQ-5D index^1^ - EQ-5D index^2^	-0.68	-0.89	-0.46
SkindexTotal^1^ - SkindexTotal^2^	0.71	0.49	0.93
Skindex Symptoms^1^ - Skindex Symptoms^2^	0.78	0.56	1.01
Skindex Emotions^1^ - Skindex Emotions^2^	0.61	0.40	0.83
Skindex Functon^1^ - Skindex Function^2^	0.59	0.37	0.80
DLQI total^1^ - DLQI total^2^	0.47	0.26	0.68
POEM^1^ - POEM^2^	0.68	0.46	0.90
BSA^1^ - BSA^2^	0.79	0.57	1.01
Pruritus VAS^1^ - Pruritus VAS^2^	0.72	0.50	0.93
EQ-VAS^1^ - EQ-VAS^2^	0.18	-0.10	0.36

^1^ Initial visit

^2^ Follow-up visit

EASI: Eczema area and severity index; SCORAD: Scoring atopic dermatitis; DLQI: Dermatology life quality index; POEM: Patient-oriented eczema measure; BSA: Body surface area; EQ-VAS: EuroQoL visual analogue scale.

Correlations between atopic dermatitis severity and quality of life scores are summarized in table 5. Significant and moderate correlations were found at the follow-up visit in most of the quality-of-life scale comparisons, and, notably, BSA and EASI correlated less with all quality-of-life instruments at the initial visit (more severe disease).

**Table 5:** Correlations between atopic dermatitis severity scores and quality-of-life scores

Atopic dermatitis severity indexes	Spearman’s correlation coefficient r (95% CI)						
	DLQI	EQ-5D index value	Skindex-29 Total	Skindex-29 Symptoms	Skindex-29 Emotions	Skindex-29 Function	EQ-VAS
BSA							
Initial Follow-up	0.17 (0, 0.33) 0.36 (0.17, 0.53)^*^	-0.14 (-0.33, -0.57) -0.39 (-0.54, -0.20)^*^	0.02 (-0.18, 0.22) 0.44 (0.27, 0.59)^*^	0.06 (-0.14, 0.25) 0.45 (0.27, 0.60)^*^	-0.03 (-0.20, 0.19) 0.40 (0.22, 0.56)^*^	-0.13 (-0.21, 0.18) 0.35 (0.16, 0.51)^*^	-0.35 (-0.49, -0.19)^*^ -0.53 (-0.66, -0.38)^*^
SCORAD							
Initial Follow-up	0.25 (0.08, 0.40)^*^ 0.46 (0.28, 0.61)^*^	-0.19 (-0.37, -0.01) -0.41 (-0.56, -0.23)^*^	0.30 (0.14, 0.45)^*^ 0.40 (0.21, 0.55)^*^	0.34 (0.18, 0.49) ^*^ 0.53 (0.37, 0.66)^*^	0.25 (0.08, 0.40)^*^ 0.36 (0.17, 0.52)^*^	0.27 (-0.10, 0.42) ^*^ 0.23 (0.03, 0.42)^*^	-0.43 (-0.56,^*^ -0.28) -0.55 (-0.68, -0.40)^*^
EASI							
Initial Follow-up	0.15 (-0.21, 0.31) 0.37 (0.17, 0.53)^*^	-0.26 (-0.42, -0.10)^*^ -0.33 (-0.50, -0.14)^*^	0.09 (-0.76, 0.26) 0.39 (0.21, 0.55)^*^	0.13 (-0.36, 0.30) 0.46 (0.29, 0.61)^*^	0.07 (-0.10, 0.23) 0.35 (0.16, 0.51)^*^	0.09 (-0.82, 0.25) 0.26 (0.06, 0.44)^*^	-0.31 (-0.45, -0.14)^*^ -0.57 (-0.69, -0.41)^*^
POEM							
Initial Follow-up	0.49 (0.35, 0.61) ^*^ 0.55 (0.40, 0.68)^*^	-0.21 (-0.37, -0.04) ^*^ -0.53 (-0.66, -0.36)^*^	0.60 (0.48, 0.70) ^*^ 0.56 (0.39, 0.68)^*^	0.70 (0.60, 0.77) ^*^ 0.79 (0.69, 0.85)^*^	0.54 (0.41,^*^ 0.65) 0.89 (0.84, 0.92)^*^	0.47 (0.33, 0.59) ^*^ 0.38 (0.19, 0.54)^*^	-0.24 (-0.42, -0.05)^*^ -0.64 (-0.75, -0.50)^*^
Pruritus VAS							
Initial Follow-up	0.09 (0.38, 0.11) 0.21 (0.04, 0.37)^*^	-0.16 (-0.35, 0.03) -0.33 (-0.47, -0.16)^*^	0.09 ( -0.11, 0.28) 0.34 (0.18, 0.48)^*^	0.23 (0.03, 0.41) ^*^ 0.35 (0.19, 0.49)^*^	-0.04 (-0.24, 0.16) 0.27 (0.10, 0.42)^*^	0.09 (-0.10, 0.29) 0.29 (0.12, 0.44)^*^	-0.47 (-0.59, -0.32)^*^ -0.53 (-0.66, -0.37)^*^

DLQI: Dermatology life quality index

^*^p<0.05

## Discussion

In this multicenter observational study, we found patients with overall moderate disease but considerable quality of life impairment at the initial visit. In addition, our results indicate that disease activity correlated better with the quality-of-life measurements when the disease was less severe. These findings differ from other studies performed in adults with atopic dermatitis that have described a good correlation between atopic dermatitis severity and the quality of life ^21^^,^^22^.

Interestingly, our study results are similar to the ones reported by Haeck *et al.*^23^ when they compared BSA (“rule of nines”) and SCORAD with DLQI as the disease improved. Like the authors of the previous study, we do not have a clear explanation for this finding. We were expecting a better correlation of disease severity indices with Skindex-29 as this scale was formally validated in our country ^24^^,^^25^ and *per se* includes items for evaluating anger, selfimage, and anxiety. However, it could be that the patient’s age is influencing these results, possibly because the adult has resigned himself more than a child or their parents to cope with the disease.

Our study included adolescent and adult patients with atopic dermatitis from several Colombian regions who were assessed in person by experienced dermatologists, assuring an in-depth characterization of the disease and an objective evaluation of the impact the disease exerts on the quality of life.

Patients' mean age was 29.1 years, a very relevant finding previously reported ^12^ not only because our population belonged to the contributive social security, meaning people with a formal job in Colombia, but also because at this age, individual productivity and labor could be more affected by any disease. We also found a female preponderance, also reported in other studies describing that atopic dermatitis is more frequent in females during adolescence and thereafter ^26^^,^^27^. Most of our patients were single, suggesting a possible interference of the disease in social relationships.

This study assessed atopic dermatitis disease severity and quality of life impairment with all the main instruments used in clinical practice or multicenter clinical trials. Disease severity was moderate which indirectly relates to the need for emollients, topical therapy, and systemic agents. When correlations of disease severity indices were evaluated, SCORAD strongly correlated with EASI (0.65), and BSA strongly correlated with SCORAD and EASI, a finding in line with Chopra *et al.*^28^, but that could be explained by the important severity of the disease in this study since EASI is considered a poorer measurer when assessing patients with more limited disease ^28^.

Regarding the quality of life, moderate to severe impairment was found at the initial visit (before starting treatment in most participants), confirming the impact the disease exerts on patients. In fact, at the follow-up visit, the Skindex-29 symptoms domain score remained severe, and the emotional domain continued to be notably affected, suggesting two scenarios: that even after the therapy, the atopic patient remains affected, or that Skindex-29 could be a better tool than the other quality of life scales since it continues showing affectation despite disease improvement.

With regard to the moderate to important magnitude of the effect at the initial of the study versus the follow-up, we found a more realistic effect of therapy among our atopic dermatitis patients as Cohen's D test does not depend on sample size ^29^. This finding suggests that treatment initiation in our patients significantly improved their quality of life and disease severity scores (table 5).

Overall, POEM showed strong correlations with the most used quality of life measurements (DLQI, EQ-5D), but particularly with the symptoms and emotions domains of Skindex-29. This finding is relevant because few studies in atopic dermatitis have reported POEM to measure the quality of life (most studies focused mainly on DLQI) ^30^. Also, POEM was recommended by the HOME International Consensus Group as the preferred assessment tool for atopic dermatitis symptoms in clinical trials ^31^.

The strengths of this study rely on a representative Colombian population sample and the fact that all outcomes were verified in person by dermatologists, diminishing the survey biases of previous studies. The limitations involved a probable selection bias because not all patients in the study completed the two visits. Statistical limitations included the scarce observation number explaining some unexpected low correlations, and correlation tests do not allow drawing conclusions about the causal relationships among the measured variables, however, from a clinical point of view, it is expected that the greater the severity of the disease, the lower the quality of life.

In conclusion, patients with atopic dermatitis experience a substantial burden in quality of life, calling for any clinician (general practitioner, pediatrician, or dermatologist) to consider this outcome in all patient evaluations in all visits. In addition, our findings indicate that disease activity correlates better with quality-of-life measurements when the disease is less severe after starting therapy, suggesting the need to evaluate the psychometric properties of some quality of life (*i.e.*, DLQI) or disease severity scales (*i.e.*, SCORAD and EASI). Although age may be influencing its occurrence, future analytical studies are required to determine the role of age and other factors besides disease activity. Finally, POEM and Skindex-29 seem to be optimal for establishing disease severity and quality of life in adults with atopic dermatitis.
